# Pre- and post- prandial appetite hormone levels in normal weight and severely obese women

**DOI:** 10.1186/1743-7075-6-32

**Published:** 2009-08-11

**Authors:** Joseph J Carlson, Amy A Turpin, Gail Wiebke, Steven C Hunt, Ted D Adams

**Affiliations:** 1 Division of Nutrition, University of Utah, HPER North Room 213, SLC, UT 84112, USA; 2 Division of Sports and Cardiovascular Nutrition, Departments of Radiology and Food Science and Human Nutrition, Michigan State University, MI 48824, USA; 3 Department of Pediatrics, Division of Medical Genetics, University of Utah, 50 N. Medical Drive Rm 2C412, SLC, UT, 84132, USA; 4 Cardiovascular Genetics Division, University of Utah, 420 Chipeta Way, Room 1160, SLC, UT, 84108, USA; 5 Intermountain Health & Fitness Institute, Division of Cardiology at LDS Hospital, 8th Avenue and C Street, SLC, UT, 84143, USA

## Abstract

**Background:**

Appetite is affected by many factors including the hormones leptin, ghrelin and adiponectin. Ghrelin stimulates hunger, leptin promotes satiety, and adiponectin affects insulin response. This study was designed to test whether the pre- and postprandial response of key appetite hormones differs in normal weight (NW) and severely obese (SO) women.

**Methods:**

Twenty three women ages 25–50 were recruited for this study including 10 NW (BMI = 23.1 ± 1.3 kg/m^2^) and 13 SO (BMI = 44.5 ± 7.1 kg/m^2^). The study was conducted in a hospital-based clinical research centre. Following a 12-hour fast, participants had a baseline blood draw, consumed a moderately high carbohydrate meal (60% carbohydrate, 20% protein, 20% fat) based on body weight. Postprandially, participants had six blood samples drawn at 0, 15, 30, 60, 90, and 120 minutes. Primary measures included pre- and post-prandial total ghrelin, leptin, adiponectin and insulin. A repeated measures general linear model was used to evaluate the hormone changes by group and time (significance p ≤ 0.05).

**Results:**

There were significant differences between the NW and the SO for all hormones in the preprandial fasting state. The postprandial responses between the SO versus NW revealed: higher leptin (p < 0.0001), lower adiponectin (p = 0.04), trend for lower ghrelin (p = 0.06) and insulin was not different (p = 0.26). Postprandial responses over time between the SO versus NW: higher leptin (p < 0.001), lower ghrelin and adiponectin (p = 0.004, p = 0.015, respectively), and trend for higher insulin (p = 0.06).

**Conclusion:**

This study indicates that significant differences in both pre- and selected post- prandial levels of leptin, ghrelin, adiponectin and insulin exist between NW and SO women. Improving our understanding of the biochemical mechanisms accounting for these differences in appetite hormones among individuals with varying body size and adiposity should aid in the development of future therapies to prevent and treat obesity.

## Background

The increasing prevalence of obesity [1-3] and subsequent obesity-related health disorders [4,5] and mortality [6,7] support the need for improved understanding of the etiology of obesity which can provide insight to promote new and effective prevention and treatment options. One important research target is appetite, a key factor influencing energy intake and regulation.

As a processing centre for appetite, the hypothalamus integrates signals from the brain, the peripheral circulation and the gastrointestinal tract to regulate energy intake and expenditure [8-11]. Within the hypothalamus, the arcuate nucleus contains peptide neurotransmitters associated with appetite [12]. Neuropeptide Y (NPY) and agouti-related peptide (AGRP) are orexigenic (induce feeding), whereas propiomelanocortin (POMC) and cocaine- and amphetamine-regulated transcript (CART) are anorexigenic (inhibit feeding) [11,13]. Neurons expressing these neuropeptides communicate with each other and with many peripheral signals including mechano- and chemo-receptors, nutrients such as glucose, amino acids and fatty acids, GI peptide hormones (i.e., cholecystokinin and ghrelin), and other hormones including insulin, leptin, and adiponectin to influence appetite level and feeding [14]. This study was designed to further explore specific appetite hormones (leptin, ghrelin, adiponectin and insulin) following a moderately high carbohydrate meal (60% carbohydrate, 20% protein, 20% fat) administered to normal weight (NW) and severely obese (SO) adult women 25–50 years of age.

This study builds upon previous adipocytokine/metabolic meal response studies by comparing two groups of women with a wide variation in body mass index (BMI). This included NW (BMI 18.5 to < 25 kg/m^2^) and SO (BMI 40–49 kg/m^2^) which is classified as stage III obesity. We hypothesized that there would be significant differences in selected appetite hormones (ghrelin, leptin, adiponectin and insulin) between NW and SO women in both the fasting pre-prandial state, and in the post-prandial state (0, 15, 30, 60, 90, 120 minutes); based on a group × time interaction effect. A secondary study aim was to evaluate if insulin or glucose levels correlated with leptin or ghrelin levels pre- and post-prandially.

## Methods

This study was conducted at the General Clinical Research Centre (GCRC) in the University of Utah Medical Centre Hospital (Salt Lake City, UT). The protocol was approved by the University of Utah IRB and the GCRC review board. All subjects signed an informed consent prior to participation. Exclusion criteria included pregnancy, recent weight-loss or participation in any dieting behaviour in the past six months, medications, smoking, menopause, and diagnoses of cancer in the past five years. The NW participants were recruited through sign postings and public radio advertisements. The SO participants were recruited from a database of individuals participating in an ongoing study of gastric bypass surgery conducted by the University of Utah Cardiovascular Genetics Division [15]. Ten women with a BMI of 21–24 kg/m^2 ^met the inclusion criteria for the NW group and consented to participate. Thirteen women met the inclusion criteria for the SO group with a BMI of 40–49 kg/m^2 ^(Stage III obesity) and consented to participate.

The NW participants were required to arrive for testing by 7:30 A.M. The SO subjects, already participating a separate obesity study [15] stayed overnight at the GCRC and began the study at the same time in the morning as the NW participants. All participants fasted for 12 hours and abstained from exercise and drinking alcohol for 24 hours prior to their visit. Height was measured using a Height-Rite Seca stadiometer (Model 225) to the nearest cm. Weight was measured using a Scaletronix scale to the nearest 0.1 kg. An RJL Systems Quantum II was used to measure body composition via bioelectrical impedance (BIA). Subjects completed a 61-item Willett food frequency questionnaire, and a 7-item physical activity questionnaire.

The study breakfast contained approximately one-quarter of the subjects kilocalorie (Kcal) needs estimated by the Harris-Benedict equation [16] adjusted body weight [(actual weight – ideal body weight) × 0.25]. The calculation and preparation of all meals was directed by the GCRC head Registered Dietitian (RD). The breakfast composition, as a percent of totals Kcals was 60% carbohydrate, 20% protein and 20% fat and contained the same food components for all participants with only the amounts varying depending on their Kcal needs (Additional file 1). Participants were asked to consume the entire meal. Any remaining food was weighed and quantified by the GCRC RD. The clinic protocol and timing are summarized in Additional file 2.

Blood measures included a fasting pre-prandial venous blood sample and six postprandial samples taken during a two-hour period following the ingestion of a breakfast meal (0, 15. 30, 60, 90 and 120 minutes). These blood samples were analyzed to evaluate changes in the levels of insulin, total ghrelin, leptin, and adiponectin. At each of the blood draws, 2 × 7 ml tubes of blood (1 serum, 1 EDTA) were used. The analysis of total ghrelin, leptin, and adiponectin were done in duplicate using RIA kits (Linco Research Inc., St. Charles, MO). Insulin was measured by an RIA kit (Coat-A-Count, DPC, Los Angeles, CA). Additionally, glucose was measured enzymatically using standard procedures. The samples were analyzed by ARUP (University of Utah Medical Centre Clinical Chemistry Lab, Salt Lake City, Utah).

### Statistical Methods

Descriptive statistics were run on all measures and results are listed as mean ± standard deviation. The primary dependent variables in this study included the hormones insulin, leptin, total ghrelin, and adiponectin. The independent variable was the study group (NW or SO). Analysis of covariance was used to test for baseline group differences in each of the hormones while controlling for age. Paired t-tests were used to determine changes from baseline across time for each individual hormone. A repeated measures general linear model was fit to the data to test for mean differences in hormone levels between groups across time. It was also used to test for dietary response differences between groups by including an interaction term between the NW and the SO ground and the various time points for the hormone measurements. To evaluate our secondary aims, Pearson correlations were used to determine if insulin or glucose levels were correlated with leptin or ghrelin levels at all time points measured. All statistics were computed with SPSS version 13 computer software (Chicago, IL). Significance was set at p ≤ 0.05.

## Results

Physical characteristics of all participants are listed in Additional file 3. Participants by group were similar in age and height, but as expected, based on the BMI inclusion criteria, their % body fat, and waist- and hip- circumference were significantly different. All participants completed the entire meal, with the exception of four SO and one NW participants. After accounting for returned food, caloric intake range was 567.1 to 803.8 Kcals (mean 720.8 ± 77.4 Kcals) in the NW group and 101.2 to 706.2 Kcals (mean 588.5 ± 176.1 Kcals) in the SO group. Kilocalorie intake was significantly less in the SO women than in the NW women (*p *= 0.038).

As hypothesized, controlling for age, there were significant differences between the NW and SO groups for baseline leptin, ghrelin and adiponectin levels. Baseline leptin levels were significantly higher in the SO group (48.1 ± 16.2 ng/ml) than in the NW group (10.1 ± 4.5 ng/ml) (*p *< 0.001); baseline ghrelin levels were significantly higher in the NW group (1418 ± 232 pg/ml) than in the SO group (1087 ± 187 pg/ml) (*p *= 0.001); and baseline adiponectin levels were significantly higher in the NW group (12.8 ± 0.6 ng/ml) than in the SO group (10.7 ± 1.7 ng/ml) (*p *< 0.001). Although baseline insulin levels were higher in the SO group (11.4 ± 6.3 μU/ml) than in the NW group (6.5 ± 8.7 μU/ml), when controlling for age these differences were not significant (*p *= 0.18).

**Figure 1:**
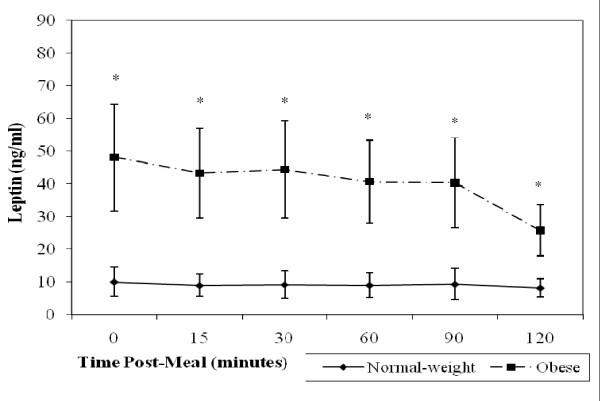
**Mean leptin levels (ng/ml) in 10 normal weight (solid line) versus 13 severely obese (broken line) participants**. The leptin response postprandially was significantly different between groups (p < 0.001), and averaged over time. Also, the severely obese participants had significantly higher *(p < 0.001) leptin levels than the normal weight participants at all measured time points.

It was hypothesized postprandial leptin would not change in the SO group, but would decrease in the NW group. Postprandially, leptin levels in the NW women decreased slowly, but this change did not reach significance until 120 minutes following the meal (*p *= 0.048 versus baseline) (Figure 1). However, in the SO women, leptin levels dropped much more dramatically than in their NW counterparts (Figure 1). These decreases were significant at 15 (*p *= 0.034), 60 (*p *= 0.019), 90 (*p *= 0.004) and 120 min (*p *= 0.002) postprandially. Compared to baseline, there was a significant group × time interaction effect for leptin postprandial responses (*p *< 0.001), indicating that leptin decreased more over time in the SO compared to the NW women. Averaged over time, the SO participants had a significantly higher postprandial leptin levels than the NW participants (*p *< 0.001). Paired student's T-tests revealed the difference between groups was significant at all measured time points (*p *< 0.001).

**Figure 2:**
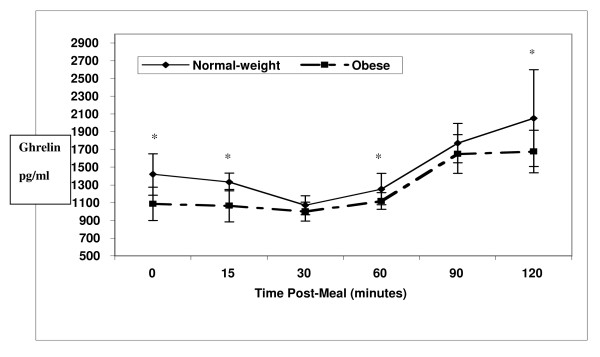
**Mean ghrelin levels (pg/ml) in 10 normal weight (solid line) versus 13 severely obese (broken line) women**. The ghrelin response postprandially showed a trend for a difference between groups (*p *= 0.06). Averaged over time, the normal weight group had significantly higher postprandial ghrelin levels than the severely obese group (*p *= 0.004). Ghrelin levels were significantly higher * (p ≤ 0.05) in normal weight women at baseline (p = 0.001), 15 (p = 0.001), 60 (p = 0.032) and 120 (p = 0.044) minutes postprandially.

We hypothesized that postprandial levels of ghrelin would decrease immediately in both groups and then increase. In the NW group, ghrelin levels decreased until 30 minutes post-meal (Figure 2). Levels began to increase at the 60-minute time point, but remained below the baseline level. Between 60 and 120 minutes following the meal, ghrelin levels became significantly higher than baseline. Compared with baseline, these changes were significant at 30 (p < 0.001), 60 (p = 0.02), 90 (p = 0.008) and 120 minutes (p = 0.002).

Ghrelin levels in the SO group decreased insignificantly from baseline until 30 minutes following the meal, then increased dramatically through 120 minutes (Figure 2). The increases reached significance at the 90- and 120-minute time-points (*p *< 0.001, for both). The ghrelin response to a meal was quadratic, and showed a trend for different response patterns between groups (*p *= 0.062). Averaged over time, the NW group had a significantly greater ghrelin response than the SO group (*p *= 0.004). Paired student's T-tests showed ghrelin levels significantly higher in NW women at baseline (*p *= 0.001), 15 (*p *= 0.001), 60 (*p *= 0.032) and 120 (*p *= 0.044) minutes post-meal compared to SO women.

**Figure 3:**
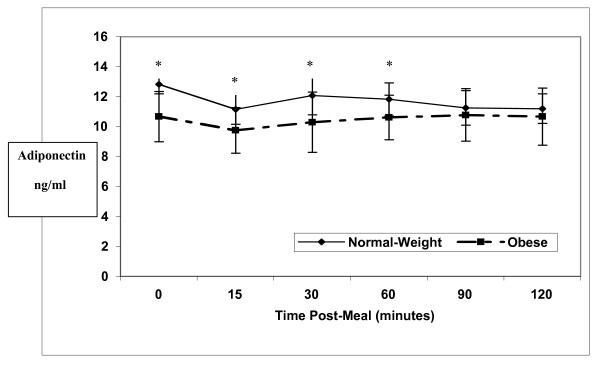
**Mean adiponectin levels (ng/ml) in 10 normal weight (solid line) versus 13 severely obese (broken line) women**. The postprandial response was significantly different between groups (*p *= 0.040), and averaged over time, the adiponectin level was significantly higher in the normal weight than in the severely obese (p = 0.015). These levels were significantly higher * (p ≤ 0.05) in normal weight women at baseline (p = 0.001), 15 (p = 0.018), 30 (p = 0.021), and 60 (p = 0.040) minutes.

In both groups, adiponectin levels decreased slightly 15 minutes postprandially and remained below baseline levels throughout the duration of the study (Figure 3). Although these changes were small, in the NW group they were significantly lower than baseline at 15 (*p *= 0.006), 60 (*p *= 0.035), 90 (*p *= 0.009) and 120 min (*p *= 0.003) following the meal. The postprandial drop in adiponectin levels in the SO group was not significant at any of the time points. The postprandial response was significantly different between groups (*p *= 0.040), and averaged over time, the adiponectin levels were significantly higher in the NW than in the SO women (*p *= 0.015). Paired student's T- tests found that these levels were significantly higher in NW women at baseline (*p *= 0.001), 15 (*p *= 0.018), 30 (*p *= 0.021), and 60 (*p *= 0.040) minutes postprandially.

**Figure 4:**
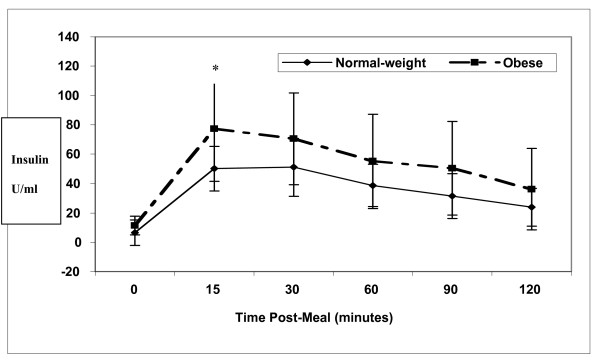
**Mean insulin levels (μU/ml) in 10 normal weight (solid line) versus 13 severely obese (broken line) participants**. The postprandial response was not significantly different between groups (*p *= 0.260), but averaged over time, insulin levels showed a trend to be higher in the severely obese group than in the normal weight group (*p *= 0.064). Insulin levels were significantly higher * (p < 0.05) in the severely obese group only at 15 minutes postprandially (p = 0.025).

Insulin levels increased dramatically following the meal, and slowly decreased through 120 minutes, but did not return to baseline levels in either group (Figure 4). In the NW and SO groups, respectively, these changes were significantly different from baseline at 15 (*p *< 0.001 for both groups), 30 (*p *< 0.001 for both groups), 60 (*p *< 0.001 and *p *= 0.001), 90 (*p *< 0.001 and *p *= 0.002) and 120 minutes (*p *= 0.002 and *p *= 0.013). As with the other hormones, we hypothesized there would be a significant group × time interaction effect with insulin. However, the postprandial response was not significantly different between groups (*p *= 0.26). Averaged over time, the mean insulin levels showed a trend to be higher in the SO group than in the NW group (*p *= 0.06). Insulin levels were significantly higher in the SO group than in the NW group only at 15 minutes postprandially (*p *= 0.025).

As a secondary aim, we evaluated the correlation of insulin and glucose levels with leptin and ghrelin levels. There was a significant correlation between insulin and leptin at 15 (*r *= 0.74, *p *= 0.015) and 30 (*r *= 0.67, *p *= 0.034) minutes postprandially in the NW women. In the SO women, there were no significant correlations between insulin and leptin at any of the time points measured. At 120 minutes post-meal, there was a trend toward an inverse correlation between insulin and ghrelin (*r *= -0.59, *p *= 0.07) in NW women, but there were no significant correlations at any of the other measured time points. Insulin and ghrelin were not significantly correlated at any of the time points measured in the SO women.

There was a significant inverse correlation in the SO group between baseline glucose levels and leptin at baseline (*r *= -0.59, *p *= 0.033), 30 (*r *= -0.62, *p *= 0.031), 60 (*r *= -0.66, *p *= 0.019) and 90 (*r *= -0.71, *p *= 0.007) minutes postprandially. Glucose was not significantly correlated with leptin levels in the NW women at any time. There was a significant inverse correlation between baseline glucose levels and ghrelin in the NW group at 90 minutes following the meal (*r *= -0.65, *p *= 0.043), but not at any other time points in this group or at any of the time points in the SO group.

## Discussion

The purpose of this study was to test whether different adipocytokine/metabolic responses to a meal exist among NW and SO women. Similar to previous research, baseline leptin levels were significantly greater in the SO group than in the NW group [17-20]. Because leptin is made in adipose tissue, the baseline difference between groups is to be expected, but because leptin is a satiety factor, higher levels would seem to cause a decrease in consumption, rather than the increased caloric intake usually associated with obesity. These results support the view that SO individuals most likely exhibit some sort of leptin resistance, either due to decreased leptin transport into the brain, or due to a down regulation of leptin receptors. Previous studies, exploring biochemical interactions of leptin with feeding and meal composition have shown leptin to decrease following fasting and dieting [21,22], and to increase in response to a carbohydrate versus a fat-rich meal [17,23,24].

In this study which used a moderately high carbohydrate meal, leptin levels decreased significantly in the SO women throughout the two-hours measured but did not change significantly in the NW women. These results differ from those reported by English, et al. [17] who observed that leptin levels decreased in the NW individuals and did not change significantly in the SO individuals. Results from our study more closely resemble the results found by Imbeault, et al., who reported increased leptin levels in NW men and decreased leptin levels in SO men following a unusually high fat meal (64% fat, 18% carbohydrate, and 18% protein), dramatically different from the meal and population we used [25]. Moreover, it is possible some the variation in results are due to adjusted Kcal levels that were based on the subjects body weight versus a single Kcal level for all participants. Because of the different results found in these studies, future leptin research should include a comparison of the response to a high-carbohydrate meal vs. a high-fat meal, including both NW and SO men and women.

Currently debate exists whether leptin levels are influenced more by insulin secretion or glucose uptake. This study demonstrated a significant correlation between insulin and leptin at selected time points (15 and 30 minutes postprandially) in NW women, but no correlation was reported at any time points in the SO women. Glucose levels were only measured at baseline in this study, but in the SO women there was a significant inverse correlation between baseline glucose levels and leptin levels at baseline 30, 60 and 90 minutes. Therefore, these results do appear to support the view that glucose may be more influential for leptin secretion than insulin in SO women.

Baseline ghrelin levels were significantly higher in the NW women than in the SO women, supporting previous research [17,18,26-30]. Ghrelin levels are an inverse indication of total energy balance and thus in SO individuals who are constantly in a state of positive energy balance, lower ghrelin levels are expected.

Baseline to 60 minutes following the meal, ghrelin levels decreased significantly in the NW group, after which they increased dramatically through 120 minutes. The SO group followed the same pattern, but did not reach significance until 90 min postprandially. These results also support previous findings that the ghrelin response to a meal is less pronounced in SO individuals than it is in NW individuals [17,21,27,31,32]. Our findings on the postprandial rise in ghrelin are different from a recent study that used a higher CHO meal (80%) and showed in NW and overweight men and women (BMIs ranging from 19–29 kg/m^2^) that the postprandial decrease in ghrelin remained below baseline levels for more than three hours, not returning to baseline levels until four hours postprandially[33]. Although ghrelin is better known for its effect on appetite stimulation, the drop in ghrelin following food intake is associated with satiety. Therefore, the less significant drop in ghrelin immediately postprandially in SO individuals may account for decreased satiety leading to increased intake over time in these individuals. This less significant drop in ghrelin immediately postprandially may have been influenced by the fact the total volume of Kcals consumed by the SO (mean 588.5 ± 176.1 Kcals) was significantly less (p = 0.038) as compared to the NW group (mean 720.8 ± 77.4 Kcals).

With respect to the relationship of ghrelin to insulin and glucose; there was a trend for an inverse correlation between insulin and ghrelin (r = -0.59, p = 0.07) in the NW group only and only at two hours postprandially. This likely would have become statistically significant with a larger sample size. Related to this there was a significant inverse correlation between baseline glucose levels and ghrelin in the NW group at 90 minutes following the meal (r = -0.65, p = 0.043). In contrast to these findings, a recent study that included 29 normal weight and overweight males and females found no relationships between ghrelin, insulin, and glucose parameters despite measurements going out six hours postprandially [33].

Like leptin, adiponectin is also made in the adipose tissue, but levels of this hormone were significantly lower in the SO group than in the NW group. These results are consistent with previous research [18,34-39], but continue to be counterintuitive. Expression of the mRNA responsible for the production of adiponectin is significantly decreased in the adipose tissue of obese mice and humans, which may explain why this occurs [35,40].

Following the meal, adiponectin levels decreased and remained below baseline levels at all measurement points. These changes were significant for the NW group, but not for the SO group, probably due to a high level of variability in the SO women. Similar results for the NW group, but very different results in the SO group were reported by English, et al. who used a similar dietary composition (56.8% carbohydrate, 12.2% protein, 31% fat) [41]. In that study, adiponectin levels rose four-fold by 60 minutes postprandially in SO individuals. The authors stated that adiponectin levels may have been affected by interference with the assay due to "exaggerated postprandial hypertriglyceridemia," but that it appears unlikely this would specifically affect the obese group. Additionally the postprandial peak in triglycerides generally occurs later than 60 minutes. Postprandial triglyceride levels of participants in the current study are not available, so we are unable to determine if exaggerated postprandial hypertriglyceridemia may have affected the results. Further research in this area is needed to determine which postprandial adiponectin profile should be expected in SO individuals.

A strength of this study was that all participants were seen in the GCRC for standardized study protocol administration. Also, the use of a body weight specific caloric load was another study strength. Previous similar hormonal feeding studies typically used a set meal size for all participants in the study which has the potential of producing alterations in hormonal responses and other blood measures (glucose) due to variations in the total substrate and Kcal load in relation to body size. Estimating the caloric needs of each participant in the current study using a widely accepted formula (Harris Benedict) may have facilitated a closer matching of each participant's actual needs.

Related to this, meal size may also have represented a limitation of this study since five participants did not finish their meals (four from the SO group and one from the NW group), which may have affected their hormone responses since the overall Kcal intake was slightly lower in the SO group. Based on appetite hormone levels differences by group, this makes sense acutely, given the SO pre- and post- prandial ghrelin levels were lower than the NW participants. In addition, several participants who did finish their meal reported the meal was larger than what they would normally consume for breakfast. A measurement limitation of this study was that our ghrelin measurement only included total ghrelin and did not include plasma acyl and desacyl ghrelin which have potential important roles in the postprandial regulation of satiety [42]. Furthermore, there are numerous other gut peptides that influence energy intake that were not measured in this study (eg. CCK, peptide YY, glucagon-like peptide-1, pancreatic polypeptide, and oxyntomodulin.) and other parameters which could have helped in explaining the results (e.g. blood lipids including triglycerides). Another limitation of this study was sample size. Although similar to the sample sizes of previous appetite hormone studies, only moderate to large changes or differences could be detected in the analyses of this study.

## Conclusion

In conclusion, both the baseline levels (fasting pre-prandial) and the postprandial response of hormones associated with appetite (ghrelin, adiponectin, and leptin) are significantly different in NW and SO women. Few studies to date have reported these responses in SO women. Since appetite is a key factor related to body composition, the hormonal differences following a meal as reported in this study may be partially responsible for weight maintenance, weight gain, or resistance to weight loss among individuals represented by different BMI classifications (i.e., NW versus SO). Further understanding of the biochemical mechanisms accounting for pre- and post- prandial hormonal variation among individuals with various body sizes and body compositions should aid in the development of therapies to prevent and treat obesity.

## Competing interests

The authors declare that they have no competing interests.

## Authors' contributions

SCH, TDA, JJC, AAT, established methods, including measures, questionnaires and procedures. GW's primary role was developing and implementing the dietary protocol with assistance from AAT and JJC. TDA oversaw and directed all aspects of this clinical research centre study and SCH oversaw all aspects of the hormone processing and analyses. AAT played a key role in the literature review. Data collection was conducted by AAT and GW with assistance from JJC. SCH lead the statistical analyses and interpretation of results. All authors participated in the writing of the paper and provided comments on the drafts and approved the final version.

## Supplementary Material

Additional file 1 Study Meal Foods and Macro-Composition*. Click here for file

Additional file 2 **Clinic Protocol.** Click here for file

Additional file 3 **Physical Characteristics of the Study Population (mean ± SD).** Click here for file
