# Supplementary material for: Pre- and post- prandial appetite hormone levels in normal weight and severely obese women
Source: Nutr Metab (Lond). 2009 Aug 11;6:32. doi: 10.1186/1743-7075-6-32 (PMC2731765; doi:10.1186/1743-7075-6-32)
Supplement: Additional file 1 — Study Meal Foods and Macro-Composition*. [file 1743-7075-6-32-S1.pdf]

Additional file 1. Study Meal Foods and Macro-Composition*

Food Item	Amount
Large Fresh Whole Egg	100 g
Margarine	1 tsp
Enriched White Bread	50 g
Jelly	12 g
Yogurt (Fruit on the Bottom)	6 oz
Orange Juice	6 oz
Skim Milk	8 oz

* Sample menu for meal providing 649 Kcals (60% carbohydrate, 20% fat, 20% protein). Amounts of eggs, milk, and juice were adjusted to account for calculated caloric needs based on the Harris Benedict equation. Actual weight was used for normal weight subjects, and adjusted body weight [(actual weight - ideal body weight) x 0.25] was used for severely obese subjects.
