# Supplementary material for: Pre- and post- prandial appetite hormone levels in normal weight and severely obese women
Source: Nutr Metab (Lond). 2009 Aug 11;6:32. doi: 10.1186/1743-7075-6-32 (PMC2731765; doi:10.1186/1743-7075-6-32)
Supplement: Additional file 2 — Clinic Protocol. [file 1743-7075-6-32-S2.pdf]

Additional file 2. Clinic Protocol

Procedure	Time
Starting time of study	7:30 am
Consent form	7:30 am
Height, weight, waist & hip circumferences,<br>3 sitting blood pressures & heart rates measured	7:45 am
Physician Admit	Before 8:00 am
5 min of rest for supine blood pressure and BIA	8:00 am
Insert cannula in back of hand/wrist and baseline blood draw	8:10 am
Meal	8:15-8:30 am
Blood drawn at 15, 30, 60, 90, and 120 min following the meal	8:45, 9:00, 9:30<br>10:00 and 10:30 am
Review questionnaires and exit interview prior to final blood draw	Leave at 10:30 am
**Total time**	**180 min (3 h)**
