# Supplementary material for: Pre- and post- prandial appetite hormone levels in normal weight and severely obese women
Source: Nutr Metab (Lond). 2009 Aug 11;6:32. doi: 10.1186/1743-7075-6-32 (PMC2731765; doi:10.1186/1743-7075-6-32)
Supplement: Additional file 3 — Physical Characteristics of the Study Population (mean ± SD). [file 1743-7075-6-32-S3.pdf]

Additional file 3. Physical Characteristics of the Study Population (mean ± SD)

Parameter	Normal Weight Women n=10	Severely Obese Women n=13
Age (y)	32.2 ± 8.6	35.6 ± 9.7
Weight (kg)	62.9 ± 4.6	124.0 ± 21.5 *
Height (cm)	164.8 ± 6.52	166.7 ± 4.0
BMI (kg/m^2)	23.1 ± 1.3	44.5 ± 7.1 *
Waist Circumference (cm)	74.8 ± 4.8	130.7 ± 17.8 *
Hip Circumference (cm)	99.7 ± 5.0	141.2 ± 15.5 *
% Body Fat	30.6 ± 3.4	56.6 ± 6.0 *
Sitting Systolic BP (mmHg)	109.5 ± 11.0	127.0 ± 16.9
Sitting Diastolic BP (mmHg)	69.2 ± 7.6	67.9 ± 10.3
Supine Systolic BP (mmHg)	106.1 ± 8.6	123.8 ± 15.4
Supine Diastolic BP (mmHg)	64.9 ± 6.7	64.3 ± 8.9

* significantly different between groups $p \leq 0.05$
